# Prognostic significance of the rho GTPase RHOV and its role in tumor immune cell infiltration: a comprehensive pan‐cancer analysis

**DOI:** 10.1002/2211-5463.13698

**Published:** 2023-08-28

**Authors:** Qin Qin, Bing Peng

**Affiliations:** ^1^ Department of Oncology Jingzhou Hospital Affiliated to Yangtze University China; ^2^ Department of Oncology The Second People's Hospital of Jingmen China

**Keywords:** immune infiltration, pan‐cancer, prognosis, RHOV, TCGA

## Abstract

Ras homolog gene family member V (RHOV) is an atypical Rho GTPase that participates in various important cellular processes. Although *RHOV* has been identified to play an oncogenic role in lung cancer and triple‐negative breast cancer, its role in other types of tumors remains unknown. In this study, we investigated the expression of *RHOV* in pan‐cancer analysis using The Cancer Genome Atlas (TCGA) and Gene‐Tissue Expression datasets. *RHOV* mRNA levels were dysregulated in several types of tumors. *RHOV* expression was identified as an independent prognostic factor in 7 of 33 types of tumors; however, the relationship varied according to tumor type. Higher *RHOV* expression was associated with a favorable prognosis in kidney renal cell carcinoma and prostate adenocarcinoma, for which *RHOV* expression was downregulated, whereas *RHOV* expression was associated with a poor prognosis for patients with adenoid cystic carcinoma, lung adenocarcinoma, pancreatic ductal adenocarcinoma, skin cutaneous melanoma, and uveal melanoma with upregulated *RHOV* expression. Furthermore, *RHOV* expression was associated with various clinicopathological parameters in these tumors. *RHOV* expression showed varied associations with different types of tumor‐infiltrating immune cells and demonstrated a potential impact on the response to immunotherapy depending on the cancer type. Additionally, functional enrichment analysis of *RHOV*‐related genes demonstrated a role in a wide range of developmental and immune‐related processes. This study provides valuable insights into the role of RHOV in pan‐cancer development, indicating its role as a tumor suppressor or oncogene according to the cancer type and tumor microenvironment.

## Graphical abstract

Pan‐cancer analysis uncovered the potential roles of RHOV. For example, in lung adenocarcinoma (LUAD), RHOV expression was upregulated, high expression correlated with worse survival and less infiltrating immune cells, indicting RHOV as an oncogene. However, in kidney renal clear cell carcinoma (KIRC), RHOV expression was downregulated, high expression correlated with better survival and more infiltrating immune cells, indicting RHOV as a tumor suppressor.

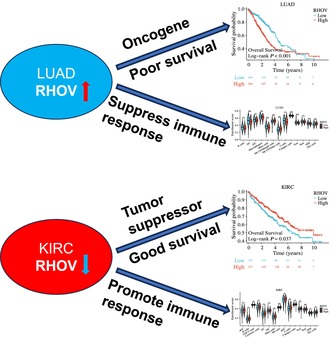

**Abbreviations**
ACC: adrenocortical carcinoma
DC: dendritic cell
EGFR: epidermal growth factor receptor
GEO: Gene Expression Omnibus
GO: Gene Ontology
GSEA: gene set enrichment analysis
GTEx: gene‐tissue expression
HPA: human protein atlas
ICI: immune checkpoint inhibitor
KEGG: Kyoto Encyclopedia of Genes and Genomes
KIRC: kidney renal clear cell carcinoma
LUAD: lung adenocarcinoma
NK: natural killer
OS: overall survival
PAAD: pancreatic adenocarcinoma
PRAD: prostate adenocarcinoma
RHOV: Ras homolog gene family member V
SKCM: skin cutaneous melanoma
TCGA: The Cancer Genome Atlas
TIDE: tumor immune dysfunction and exclusion
TIIC: tumor‐infiltrating immune cell
TIMER: tumor immune estimation resource
TME: tumor microenvironment
TNBC: triple‐negative breast cancer
UVM: uveal melanoma

The tumor microenvironment (TME) is an intricate, dynamic, and cell‐rich environment that plays a critical role in cancer initiation, progression, and treatment response [1, 2]. The TME consists of a diverse array of cells, including immune cells and stromal cells, along with extracellular matrix, blood, and lymphatic vessels [3]. Tumor‐infiltrating immune cells (TIICs), including T cells, B cells, natural killer (NK) cells, tumor‐associated macrophages, dendritic cells (DCs), and myeloid‐derived suppressor cells, play an important role in establishing the TME to facilitate tumor growth and survival [3].

Immune checkpoint inhibitors (ICIs) have revolutionized cancer treatment over the past decade, resulting in remarkable outcomes in some patients [4]. The efficacy of ICIs is influenced by various factors, including the tumor mutation burden, expression of programmed cell death‐1 and its ligand, and characteristics of the TME [1]. The proportion and distribution of TIICs also play roles in ICI efficacy [5]. Therefore, TIICs have attracted considerable research attention in recent years because they shape the immune response against cancer [6]. In particular, understanding the characteristics of TIICs and their relationship with tumor‐specific oncogenic alterations has become an important focus in data mining research [7, 8, 9, 10, 11, 12, 13].

The Rho family of GTPases, which belongs to the superfamily of Ras‐related small GTPases, comprises 20 members that can be categorized as classical or atypical Rho GTPases [14]. In general, Rho GTPases switch between an inactive and active GTP‐bound forms regulated by the balance between guanine nucleotide exchange factors and GTPase‐activating proteins [15]. Rho GTPases participate in various important cellular processes, including cell migration, adhesion, division, and cytoskeleton formation [16]. Ras homolog gene family member V (RHOV) is an atypical Rho GTPase that has been implicated in apoptosis, cell differentiation, migration, and cell–cell adhesion [17, 18, 19, 20]. Ras homolog gene family member V is also overexpressed in certain cancers such as lung cancer and triple‐negative breast cancer (TNBC), suggesting a potential oncogenic role with prognostic significance [21, 22, 23, 24]. However, a comprehensive pan‐cancer analysis of RHOV across different cancer types is lacking.

With the convenience of public databases such as The Cancer Genome Atlas (TCGA) and Gene Expression Omnibus (GEO), numerous researchers have investigated the association between specific genes and tumor immune infiltration, aiming to predict the efficacy of ICIs [8, 9, 10, 12, 25]. As RHOV participates in the differentiation of myeloid cells, especially along the monocyte lineage [26], we hypothesized that RHOV expression might correlate with TIICs. Therefore, in this study, we performed a comprehensive bioinformatic analysis to investigate the prognostic significance of *RHOV* expression in pan‐cancer. We also investigated the relationship between *RHOV* expression, clinicopathological parameters, TIICs, and the response to ICIs. This analysis can provide further insight into the potential biological functions of RHOV as a candidate target for new pan‐cancer immunotherapies and treatment strategies (Fig. 1).

**Fig. 1:**
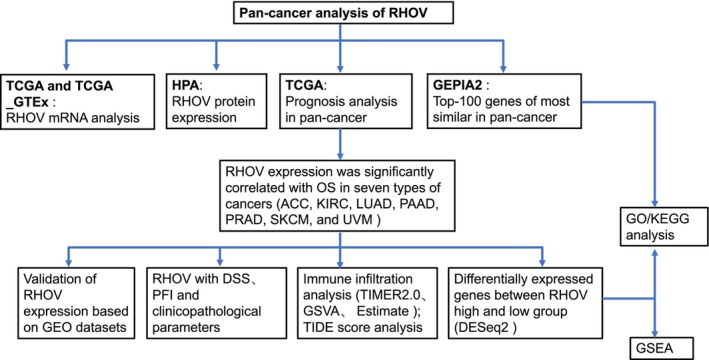
The workflow diagram of the study.

## Materials and methods

### *RHOV* expression pattern in human pan‐cancer analysis

Clinical and gene expression data were derived from tumor and paraneoplastic tissues for 33 tumor types from tcga (https://portal.gdc.cancer.gov/) portal. Considering the small number of control samples in TCGA database and the lack of corresponding control samples from adrenocortical carcinoma (ACC), diffuse large B‐cell lymphoma, acute myeloid leukemia, low‐grade glioma, mesothelioma, ovarian serous cystadenocarcinoma, testicular germ cell tumors, uterine carcinosarcoma, and uveal melanoma (UVM), we supplemented normal tissue data from TCGA_Gene‐Tissue Expression (GTEx) datasets obtained from the University of California Santa Cruz xena portal (https://xenabrowser.net/datapages/). However, no normal group was available for mesothelioma and UVM in either database. Data of the normal control group for cholangiocarcinoma, head and neck squamous cell carcinoma, pheochromocytoma and paraganglioma, and sarcoma were obtained from TCGA only. For other cancer types, data of the normal control group were obtained from both TCGA and GTEx databases. Both databases were used to explore the potential dysregulation of *RHOV* expression between all 33 tumor types and normal tissues; TCGA dataset was used to compare the differential expression of *RHOV* between 23 types of tumors and matched normal tissues. Gene expression levels were represented as log2 (TPM + 1) values.

The Human Protein Atlas (HPA; https://www.proteinatlas.org/) was used to determine the protein expression of RHOV in 44 different normal tissues and 20 types of tumors based on immunohistochemical images and corresponding analysis.

### Prognosis analysis

Univariate and multivariate Cox proportional hazards regression analyses were used to identify whether *RHOV* expression was an independent prognostic factor for overall survival (OS) in patients with 33 cancer types from TCGA. The associations of *RHOV* expression with disease‐specific survival and the progression‐free interval were further investigated in cancers showing a significant association of *RHOV* expression with OS. Patients were dichotomized into low‐ and high‐expression groups based on median *RHOV* mRNA expression level. Forest plots and Kaplan–Meier survival curves were used to visualize the results. Hazard ratios and 95% confidence intervals were calculated using univariate survival analysis, and the survival curves with *P* < 0.05.

### Independent validation of *RHOV* expression based on GEO datasets

To further verify the differential expression of *RHOV* in tumors showing a significant correlation of *RHOV* with prognosis, we compared gene expression profiles of normal and tumor samples from the GEO datasets (https://www.ncbi.nlm.nih.gov/geo/) GSE10927 (ACC), GSE36895 (kidney renal clear cell carcinoma [KIRC]), GSE53757 (KIRC), GSE31210 (lung adenocarcinoma [LUAD]), GSE71729 (pancreatic adenocarcinoma [PAAD]), GSE32571 (prostate adenocarcinoma [PRAD]), and GSE15605 (skin cutaneous melanoma [SKCM]).

### Correlation between RHOV expression and clinicopathological parameters

The correlation between RHOV expression and available clinicopathological parameters, including pathologic/clinical stage, T stage, N stage, M stage, treatment outcomes, and histological grade, was evaluated in tumors for which *RHOV* expression was significantly associated with prognosis. In addition, prostate‐specific antigen and Gleason scores were included for the PRAD analysis, whereas ulceration and Breslow depth were included for the SKCM analysis.

### Immune infiltration analysis and prediction of ICI response

Tumor Immune Estimation Resource 2.0 (timer 2.0; http://timer.cistrome.org/) was used to analyze the correlation between *RHOV* expression and various types of immune cells, including CD4+ T cells, CD8+ T cells, B cells, macrophages, NK cells, and regulatory T cells, across the seven tumor types showing a significant prognostic association with *RHOV* in TCGA [27]. Single‐sample gene set enrichment analysis (GSEA) was further employed using the r package ‘GSVA’ to measure the per‐sample infiltration levels of 24 immune cell types [28]. Finally, the r package ‘estimate’ was used to calculate the ImmuneScore, StromalScore, and ESTIMATEScore in the TME [29].

The Tumor Immune Dysfunction and Exclusion (TIDE) algorithm was used to predict the potential benefits of ICIs for patients according to the TIICs analysis [30]. In addition, the IMvigor210 cohort was examined to investigate the potential role of RHOV in predicting the benefits of ICIs [31].

### Functional enrichment analysis

Gene Ontology (GO), including biological pathway, cellular component, and molecular function terms, and Kyoto Encyclopedia of Genes and Genomes (KEGG) pathway analyses were performed based on *RHOV*‐related genes, which were selected according to the top 100 genes with similar expression patterns to those of *RHOV* from the GEPIA2 database (http://gepia2.cancer‐pku.cn/). In addition, enrichment analyses were performed according to differentially expressed genes in the high‐ and low‐*RHOV* expression groups for the seven tumors in which *RHOV* expression was associated with prognosis. Differentially expressed genes were identified using the ‘deseq2’ r package according to adjusted *P* value < 0.05 and log2 fold change ≥ 1.5 or ≤ −1.5. GSEA was also performed on these differentially expressed genes using the ‘clusterprofiler’ r package.

### Statistical analysis

The Wilcoxon rank‐sum test was used to compare differences between two groups, and the Spearman rank test was used to calculate correlations. The log‐rank test was used for Kaplan–Meier survival analysis. r version 4.2.2 was used for all bioinformatic and statistical analyses. *P* < 0.05 was considered statistically significant.

## Results

### Pan‐cancer expression landscape of *RHOV*

**Fig. 2:**
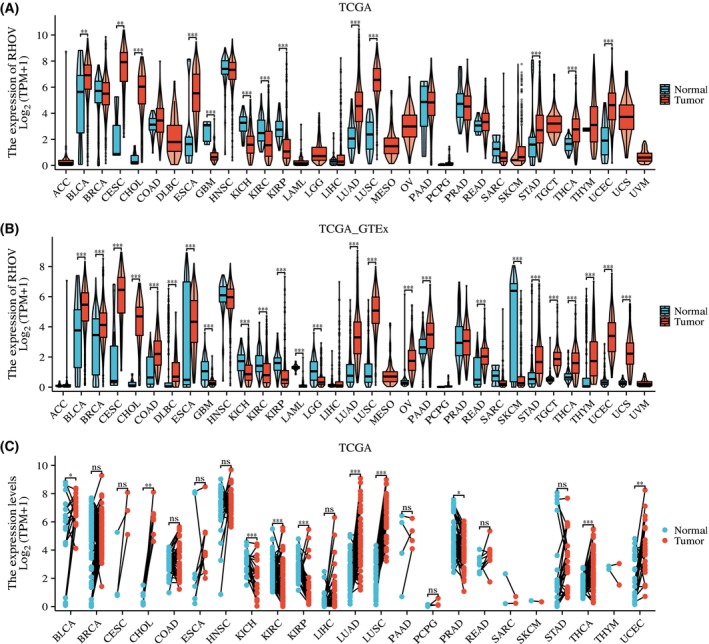
The mRNA expression of *RHOV* in pan‐cancer. (A) Comparison of the mRNA expression of *RHOV* between tumor and normal tissue in 33 tumors in The Cancer Genome Atlas (TCGA) database. ACC Tumor (*n* = 79). BLCA Normal (*n* = 19), Tumor (*n* = 412). BRCA Normal (*n* = 113), Tumor (*n* = 1113). CESC Normal (*n* = 3), Tumor (*n* = 306). CHOL Normal (*n* = 9), Tumor (*n* = 35). COAD Normal (*n* = 41), Tumor (*n* = 480). DLBC Tumor (*n* = 48). ESCA Normal (*n* = 11), Tumor (*n* = 163). GBM Normal (*n* = 5), Tumor (*n* = 169). HNSC Normal (*n* = 44), Tumor (*n* = 504). KICH Normal (*n* = 25), Tumor (*n* = 65). KIRC Normal (*n* = 72), Tumor (*n* = 541). KIRP Normal (*n* = 32), Tumor (*n* = 291). LAML Tumor (*n* = 150). LGG Tumor (*n* = 532). LIHC Normal (*n* = 50), Tumor (*n* = 374). LUAD Normal (*n* = 59), Tumor (*n* = 539). LUSC Normal (*n* = 49), Tumor (*n* = 502). MESO Tumor (*n* = 87). OV Tumor (*n* = 381). PAAD Normal (*n* = 4), Tumor (*n* = 179). PCPG Normal (*n* = 3), Tumor (*n* = 184). PRAD Normal (*n* = 52), Tumor (*n* = 501). READ Normal (*n* = 10), Tumor (*n* = 167). SARC Normal (*n* = 2), Tumor (*n* = 263). SKCM Normal (*n* = 1), Tumor (*n* = 472). STAD Normal (*n* = 32), Tumor (*n* = 375). TGCT Tumor (*n* = 156). THCA Normal (*n* = 59), Tumor (*n* = 512). THYM Normal (*n* = 2), Tumor (*n* = 120). UCEC Normal (*n* = 35), Tumor (*n* = 554). UCS Tumor (*n* = 57). UVM Tumor (*n* = 80). (B) Comparison of mRNA expression of *RHOV* between tumor and normal tissue in 33 tumors in TCGA and Gene‐Tissue Expression (GTEx) database. ACC Normal (*n* = 128), Tumor (*n* = 77). BLCA Normal (*n* = 28), Tumor (*n* = 407). BRCA Normal (*n* = 292), Tumor (*n* = 1099). CESC Normal (*n* = 13), Tumor (*n* = 306). CHOL Normal (*n* = 9), Tumor (*n* = 36). COAD Normal (*n* = 349), Tumor (*n* = 290). DLBC Normal (*n* = 444), Tumor (*n* = 47). ESCA Normal (*n* = 666), Tumor (*n* = 182). GBM Normal (*n* = 1157), Tumor (*n* = 166). HNSC Normal (*n* = 44), Tumor (*n* = 520). KICH Normal (*n* = 53), Tumor (*n* = 66). KIRC Normal (*n* = 100), Tumor (*n* = 531). KIRP Normal (*n* = 60), Tumor (*n* = 289). LAML Normal (*n* = 70), Tumor (*n* = 173). LGG Normal (*n* = 1152), Tumor (*n* = 523). LIHC Normal (*n* = 160), Tumor (*n* = 371). LUAD Normal (*n* = 347), Tumor (*n* = 515). LUSC Normal (*n* = 338), Tumor (*n* = 498). MESO Tumor (*n* = 87). OV Normal (*n* = 88), Tumor (*n* = 427). PAAD Normal (*n* = 171), Tumor (*n* = 179). PCPG Normal (*n* = 3), Tumor (*n* = 182). PRAD Normal (*n* = 152), Tumor (*n* = 496). READ Normal (*n* = 318), Tumor (*n* = 93). SARC Normal (*n* = 2), Tumor (*n* = 262). SKCM Normal (*n* = 813), Tumor (*n* = 469). STAD Normal (*n* = 210), Tumor (*n* = 414). TGCT Normal (*n* = 165), Tumor (*n* = 154). THCA Normal (*n* = 338), Tumor (*n* = 512). THYM Normal (*n* = 446), Tumor (*n* = 119). UCEC Normal (*n* = 101), Tumor (*n* = 181). UCS Normal (*n* = 78), Tumor (*n* = 57). UVM Tumor (*n* = 79). (C) Comparison of mRNA expression of RHOV in paired normal and tumor samples for 23 tumor types available in TCGA database. BLCA (*n* = 19). BRCA (*n* = 113). CESC (*n* = 3). CHOL (*n* = 8). COAD (*n* = 41). ESCA (*n* = 8). HNSC (*n* = 43). KICH (*n* = 24). KIRC (*n* = 72). KIRP (*n* = 32). LIHC (*n* = 50). LUAD (*n* = 58). LUSC (*n* = 49). PAAD (*n* = 4). PCPG (*n* = 3). PRAD (*n* = 52). READ (*n* = 9). SARC (*n* = 2). SKCM (*n* = 1). STAD (*n* = 27). THCA (*n* = 59). THYM (*n* = 2). UCEC (*n* = 23). ACC, adrenocortical carcinoma; BLCA, bladder urothelial carcinoma; BRCA, breast invasive carcinoma; CESC, cervical squamous cell carcinoma; CHOL, cholangiocarcinoma; COAD, colon adenocarcinoma; DLBC, diffuse large b‐cell Lymphoma; ESCA, esophageal carcinoma; GBM, glioblastoma multiforme; HNSC, head and neck squamous cell carcinoma; KICH, kidney chromophobe; KIRC, kidney renal clear cell carcinoma; KIRP, kidney renal papillary cell carcinoma; LAML, acute myeloid leukemia; LGG, brain lower grade glioma; LIHC, liver hepatocellular carcinoma; LUAD, lung adenocarcinoma; LUSC, lung squamous cell carcinoma; MESO, mesothelioma; OV, ovarian serous cystadenocarcinoma; PAAD, pancreatic adenocarcinoma; PCPG, pheochromocytoma and paraganglioma; PRAD, prostate adenocarcinoma; READ, rectum adenocarcinoma; SARC, sarcoma; SKCM, skin cutaneous melanoma; STAD, stomach adenocarcinoma; TGCT, testicular germ cell tumors; THCA, thyroid carcinoma; THYM, thymoma; UCEC, uterine corpus endometrial carcinoma; UCS, uterine carcinosarcoma; UVM, uveal melanoma. (Wilcoxon rank‐sum test, ns, *P* > 0.05; *P < 0.05; **P < 0.01; ***P < 0.001).

Analyses of TCGA and TCGA_GTEx databases showed that *RHOV* mRNA levels were dysregulated in several tumors (Fig. 2). According to TCGA_GTEx analysis, *RHOV* expression was upregulated in 18 tumor types, including bladder urothelial carcinoma, breast invasive carcinoma, cervical squamous cell carcinoma and endocervical adenocarcinoma, cholangiocarcinoma, colon adenocarcinoma, diffuse large B‐cell lymphoma, esophageal carcinoma, LUAD, lung squamous cell carcinoma, ovarian serous cystadenocarcinoma, PAAD, rectum adenocarcinoma, stomach adenocarcinoma, testicular germ cell tumors, thyroid carcinoma, thymoma, uterine corpus endometrial carcinoma, and uterine carcinosarcoma. Conversely, *RHOV* expression was downregulated in seven tumor types: glioblastoma multiforme, kidney chromophobe, KIRC, kidney renal papillary cell carcinoma, acute myeloid leukemia, low‐grade glioma, and SKCM.

**Fig. 3:**
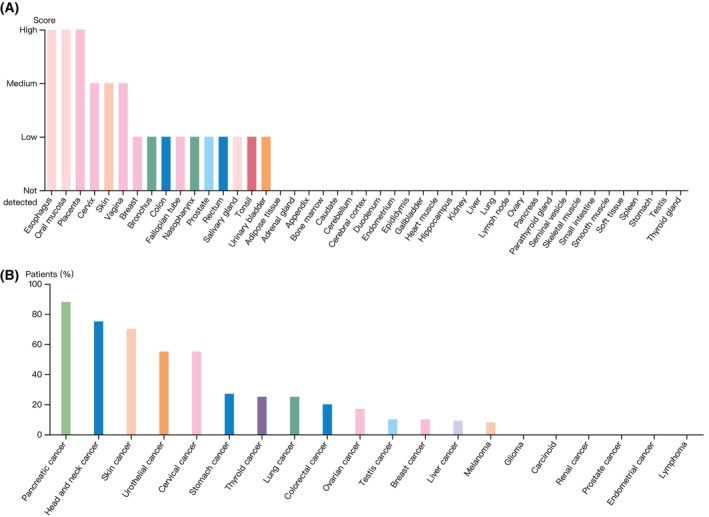
Protein expression of RHOV in normal and tumor tissues downloaded from the Human Protein Atlas database. (A) Normal tissues. (B) Tumor tissues.

**Fig. 4:**
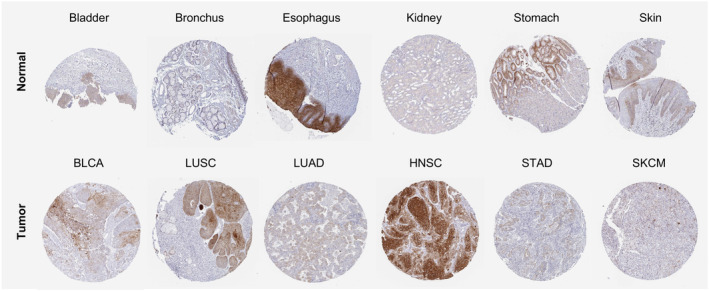
Representative immunohistochemistry images of RHOV expression in normal and tumor tissues extracted from the Human Protein Atlas database.

According to the HPA dataset, RHOV protein was expressed in several normal tissues, with the highest expression observed in the squamous epithelia such as the esophagus, oral mucosa, and skin (Fig. 3A). In terms of tumor tissues, pancreatic cancer, head and neck cancer, skin cancer, urothelial cancer, cervical cancer, and stomach cancer showed moderate to strong cytoplasmic and/or membranous staining of RHOV, whereas RHOV staining was negative for glioma, carcinoid, renal cancer, prostate cancer, endometrial cancer, and lymphoma (Fig. 3B). Representative immunohistochemistry images of RHOV protein expression in normal and tumor tissues are shown in Fig. 4.

### Association between *RHOV* expression and prognosis in pan‐cancer

**Fig. 5:**
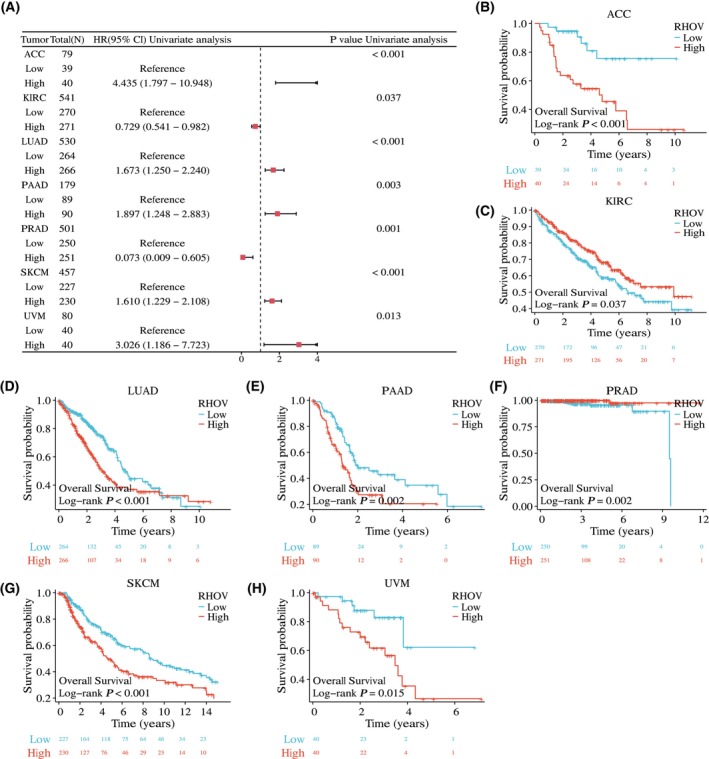
Significant association between *RHOV* expression and overall survival (OS) in seven cancer types. (A) Forest plot. (B–H) Kaplan–Meier survival curves displaying the correlation between *RHOV* expression and OS.

**Fig. 6:**
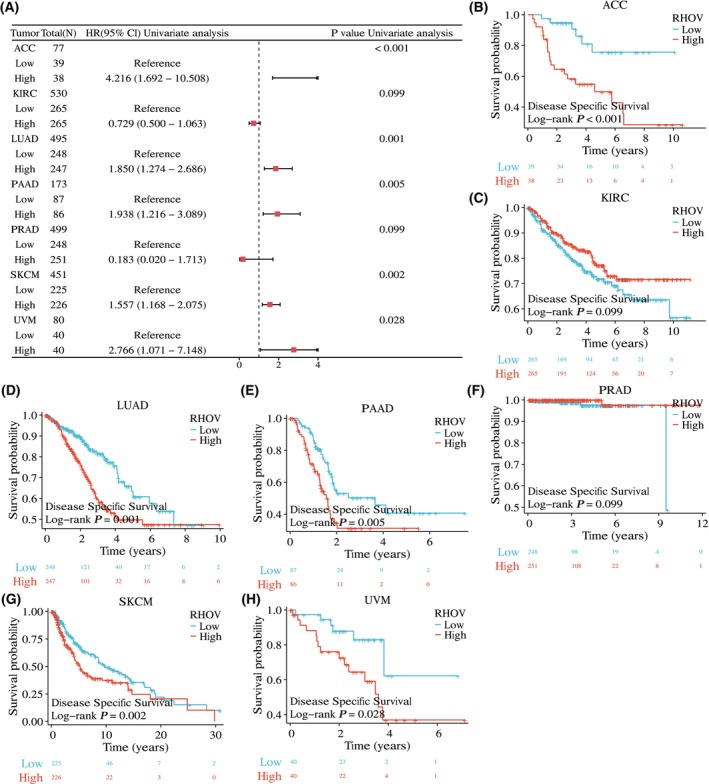
Association between RHOV expression and disease‐specific survival (DSS) in seven types of cancers. (A) Forest plot. (B–H) Kaplan–Meier survival curves displaying the correlation between RHOV expression and DSS.

**Fig. 7:**
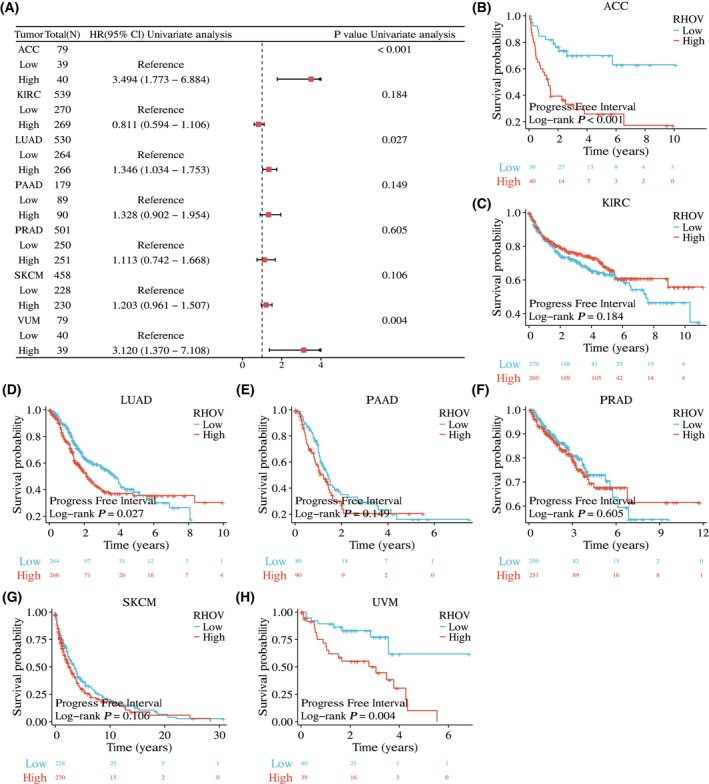
Association between RHOV expression and progression‐free interval (PFI) in seven types of cancers. (A) Forest plot. (B–H) Kaplan–Meier survival curves displaying the correlation between RHOV expression and PFI.

The univariate Cox regression analyses showed that *RHOV* expression was significantly correlated with OS in seven tumor types: ACC, KIRC, LUAD, PAAD, PRAD, SKCM, and UVM (Fig. 5A; Table 1). Kaplan–Meier survival curves demonstrated that upregulated *RHOV* expression was associated with poor OS in patients with ACC, LUAD, PAAD, SKCM, and UVM (Fig. 5B,D,E,G,H). Conversely, downregulated *RHOV* expression was associated with unfavorable OS in patients with KIRC and PRAD (Fig. 5C,F). Multivariate Cox regression analysis confirmed *RHOV* as an independent prognostic factor in these seven tumors (Tables 2, 3, 4, 5, 6, 7, 8). Furthermore, high expression levels of *RHOV* correlated with worse disease‐specific survival in patients with ACC, LUAD, PAAD, SKCM, and UVM, and were associated with a shorter progression‐free interval in patients with ACC and LUAD (Figs 6 and 7).

**Table 1:** Univariate Cox regression of RHOV expression with OS in pan‐cancer.

Cancer type	Total (*N*)	HR (95% CI)	*P* value
**ACC**	**79**	**4.435 (1.797–10.948)**	**< 0.001**
BLCA	411	1.108 (0.828–1.484)	0.490
BRCA	1086	1.154 (0.836–1.592)	0.383
CESC	306	1.328 (0.830–2.123)	0.234
CHOL	35	0.768 (0.294–2.003)	0.589
COAD	477	1.442 (0.977–2.130)	0.064
DLBC	48	3.671 (0.736–18.313)	0.086
ESCA	163	0.964 (0.590–1.577)	0.884
GBM	168	1.121 (0.799–1.573)	0.507
HNSC	503	0.830 (0.636–1.085)	0.172
KICH	64	1.320 (0.353–4.930)	0.679
**KIRC**	**541**	**0.729 (0.541–0.982)**	**0.037**
KIRP	290	1.158 (0.639–2.100)	0.628
LAML	139	0.729 (0.477–1.113)	0.142
LGG	530	0.883 (0.630–1.238)	0.471
LIHC	373	1.281 (0.907–1.809)	0.159
**LUAD**	**530**	**1.673 (1.250–2.240)**	**< 0.001**
LUSC	496	0.810 (0.617–1.064)	0.128
MESO	86	0.949 (0.597–1.508)	0.825
OV	379	1.054 (0.814–1.364)	0.69
**PAAD**	**179**	**1.897 (1.248–2.883)**	**0.003**
PCPG	184	0.618 (0.147–2.594)	0.504
**PRAD**	**501**	**0.073 (0.009–0.605)**	**0.001**
READ	166	1.919 (0.875–4.207)	0.101
SARC	263	0.998 (0.673–1.480)	0.991
**SKCM**	**457**	**1.610 (1.229–2.108)**	**< 0.001**
STAD	370	0.998 (0.720–1.385)	0.993
TGCT	139	0.237 (0.023–2.417)	0.188
THCA	512	0.486 (0.169–1.400)	0.166
THYM	119	1.611 (0.402–6.458)	0.492
UCEC	553	1.272 (0.847–1.909)	0.246
UCS	57	0.953 (0.490–1.852)	0.886
**UVM**	**80**	**3.026 (1.186–7.723)**	**0.013**

**Table 2:** Univariate and multivariate cox regression analysis for OS in ACC.

Characteristics	Total (*N*)	Univariate analysis		Multivariate analysis	
		Hazard ratio (95% CI)	*P* value	Hazard ratio (95% CI)	*P* value
Age	79		0.125		
≤ 50	41	Reference			
> 50	38	1.799 (0.846–3.824)	0.127		
Gender	79		0.999		
Female	48	Reference			
Male	31	1.001 (0.469–2.137)	0.999		
Pathologic T stage	77		**< 0.001**		
T1&T2	51	Reference		Reference	
T3&T4	26	10.286 (3.976–26.608)	**< 0.001**	11.505 (1.459–90.722)	**0.020**
Pathologic N stage	77		0.184		
N0	68	Reference			
N1	9	2.038 (0.769–5.400)	0.152		
Pathologic stage	77		**< 0.001**		
Stage I&Stage II	46	Reference		Reference	
Stage III&Stage IV	31	6.476 (2.706–15.498)	**< 0.001**	0.692 (0.084–5.678)	0.732
RHOV	79		**< 0.001**		
Low	39	Reference		Reference	
High	40	4.435 (1.797–10.948)	**0.001**	3.370 (1.327–8.560)	**0.011**

**Table 3:** Univariate and multivariate cox regression analysis for OS in KIRC.

Characteristics	Total (*N*)	Univariate analysis		Multivariate analysis	
		Hazard ratio (95% CI)	*P* value	Hazard ratio (95% CI)	*P* value
Age	541		**< 0.001**		
≤ 60	269	Reference		Reference	
> 60	272	1.791 (1.319–2.432)	**< 0.001**	1.717 (1.113–2.650)	**0.015**
Gender	541		0.614		
Female	187	Reference			
Male	354	0.924 (0.679–1.257)	0.613		
Pathologic T stage	541		**< 0.001**		
T1	279	Reference		Reference	
T2	71	1.490 (0.895–2.481)	0.125	0.118 (0.022–0.640)	**0.013**
T3&T4	191	3.555 (2.536–4.982)	**< 0.001**	0.443 (0.125–1.566)	0.206
Pathologic N stage	258		**0.001**		
N0	242	Reference		Reference	
N1	16	3.422 (1.817–6.446)	**< 0.001**	1.842 (0.909–3.730)	0.090
Pathologic stage	538		**< 0.001**		
Stage I	273	Reference		Reference	
Stage II	59	1.183 (0.638–2.193)	0.594	7.304 (1.106–48.255)	**0.039**
Stage III	123	2.649 (1.767–3.971)	**< 0.001**	5.317 (1.407–20.090)	**0.014**
Stage IV	83	6.622 (4.535–9.670)	**< 0.001**	20.021 (4.716–84.999)	**< 0.001**
RHOV	541		**0.037**		
Low	270	Reference		Reference	
High	271	0.729 (0.541–0.982)	**0.037**	0.479 (0.309–0.743)	**0.001**

**Table 4:** Univariate and multivariate cox regression analysis for OS in LUAD.

Characteristics	Total (*N*)	Univariate analysis		Multivariate analysis	
		Hazard ratio (95% CI)	*P* value	Hazard ratio (95% CI)	*P* value
Age	520		0.185		
≤ 65	257	Reference			
> 65	263	1.216 (0.910–1.625)	0.186		
Gender	530		0.570		
Female	283	Reference			
Male	247	1.087 (0.816–1.448)	0.569		
Pathologic T stage	527		**< 0.001**		
T1	176	Reference		Reference	
T2	285	1.507 (1.059–2.146)	**0.023**	1.269 (0.882–1.827)	0.199
T3&T4	66	3.095 (1.967–4.868)	**< 0.001**	1.882 (1.096–3.230)	**0.022**
Pathologic N stage	514		**< 0.001**		
N0	345	Reference		Reference	
N1&N2&N3	169	2.547 (1.904–3.407)	**< 0.001**	1.530 (0.895–2.615)	0.120
Pathologic stage	522		**< 0.001**		
Stage I	292	Reference		Reference	
Stage II	123	2.341 (1.638–3.346)	**< 0.001**	1.405 (0.785–2.515)	0.252
Stage III&Stage IV	107	3.635 (2.574–5.132)	**< 0.001**	1.898 (0.973–3.701)	0.060
RHOV	530		**< 0.001**		
Low	264	Reference		Reference	
High	266	1.673 (1.250–2.240)	**< 0.001**	1.375 (1.015–1.862)	**0.039**

**Table 5:** Univariate and multivariate cox regression analysis for OS in PAAD.

Characteristics	Total (*N*)	Univariate analysis		Multivariate analysis	
		Hazard ratio (95% CI)	*P* value	Hazard ratio (95% CI)	*P* value
Age	179		0.230		
≤ 65	94	Reference			
> 65	85	1.285 (0.853–1.937)	0.230		
Gender	179		0.320		
Female	80	Reference			
Male	99	0.813 (0.541–1.222)	0.319		
Pathologic T stage	177		**0.017**		
T1&T2	31	Reference		Reference	
T3&T4	146	2.035 (1.079–3.838)	**0.028**	1.576 (0.586–4.243)	0.368
Pathologic N stage	174		**0.002**		
N0	50	Reference		Reference	
N1	124	2.161 (1.287–3.627)	**0.004**	2.126 (1.129–4.005)	**0.020**
Pathologic stage	176		**0.018**		
Stage I	21	Reference		Reference	
Stage II&Stage III&Stage IV	155	2.309 (1.059–5.033)	**0.035**	0.555 (0.143–2.150)	0.395
RHOV	179		**0.003**		
Low	89	Reference		Reference	
High	90	1.897 (1.248–2.883)	**0.003**	1.766 (1.154–2.701)	**0.009**

**Table 6:** Univariate and multivariate cox regression analysis for OS in PRAD.

Characteristics	Total (*N*)	Univariate analysis		Multivariate analysis	
		Hazard ratio (95% CI)	*P* value	Hazard ratio (95% CI)	*P* value
Age	501		0.479		
≤ 60	225	Reference			
> 60	276	1.578 (0.441–5.650)	0.484		
Pathologic T stage	494		0.134		
T2	189	Reference			
T3&T4	305	3.277 (0.608–17.655)	0.167		
Pathologic N stage	428		0.126		
N0	348	Reference			
N1	80	3.470 (0.767–15.695)	0.106		
Gleason score	501		**0.007**		
6&7	294	Reference		Reference	
8&9&10	207	6.654 (1.371–32.298)	**0.019**	9.407 (1.647–53.718)	**0.012**
RHOV	501		**0.001**		
Low	250	Reference		Reference	
High	251	0.073 (0.009–0.605)	**0.015**	0.053 (0.006–0.500)	**0.010**

**Table 7:** Univariate and multivariate cox regression analysis for OS in SKCM.

Characteristics	Total (*N*)	Univariate analysis		Multivariate analysis	
		Hazard ratio (95% CI)	*P* value	Hazard ratio (95% CI)	*P* value
Age	457		**< 0.001**		
≤ 60	247	Reference		Reference	
> 60	210	1.663 (1.256–2.201)	**< 0.001**	1.242 (0.893–1.727)	0.197
Gender	457		0.256		
Female	173	Reference			
Male	284	1.180 (0.885–1.574)	0.261		
Pathologic T stage	362		**< 0.001**		
T1	42	Reference		Reference	
T2	77	1.523 (0.826–2.806)	0.178	1.710 (0.866–3.376)	0.122
T3	90	2.135 (1.179–3.867)	**0.012**	1.915 (0.975–3.761)	0.059
T4	153	3.780 (2.109–6.776)	**< 0.001**	3.794 (1.942–7.411)	**< 0.001**
Pathologic N stage	403		**< 0.001**		
N0	225	Reference		Reference	
N1&N2&N3	178	1.760 (1.310–2.365)	**< 0.001**	2.857 (1.015–8.040)	**0.047**
Pathologic stage	411		**0.001**		
Stage I&Stage II	218	Reference		Reference	
Stage III&Stage IV	193	1.625 (1.213–2.175)	**0.001**	0.683 (0.243–1.923)	0.470
RHOV	457		**< 0.001**		
Low	227	Reference		Reference	
High	230	1.610 (1.229–2.108)	**< 0.001**	1.488 (1.083–2.045)	**0.014**

**Table 8:** Univariate and multivariate cox regression analysis for OS in UVM.

Characteristics	Total (*N*)	Univariate analysis		Multivariate analysis	
		Hazard ratio (95% CI)	*P* value	Hazard ratio (95% CI)	*P* value
Age	80		0.075		
≤ 60	40	Reference		Reference	
> 60	40	2.123 (0.914–4.933)	0.080	3.449 (1.350–8.814)	**0.010**
Gender	80		0.316		
Female	35	Reference			
Male	45	1.542 (0.651–3.652)	0.325		
Clinical T stage	78		0.051		
T2&T3	40	Reference		Reference	
T4	38	2.416 (0.964–6.055)	0.060	5.681 (1.917–16.834)	**0.002**
Clinical N stage	80		0.058		
N0	76	Reference		Reference	
NX	4	6.177 (1.302–29.304)	**0.022**	15.583 (1.594–152.382)	**0.018**
Clinical stage	80		0.220		
Stage II	36	Reference			
Stage III&Stage IV	44	1.718 (0.704–4.193)	0.234		
RHOV	80		**0.013**		
Low	40	Reference		Reference	
High	40	3.026 (1.186–7.723)	**0.021**	3.629 (1.338–9.844)	**0.011**

### Validation of *RHOV* expression patterns in ACC, KIRC, LUAD, PAAD, PRAD, and SKCM

**Fig. 8:**
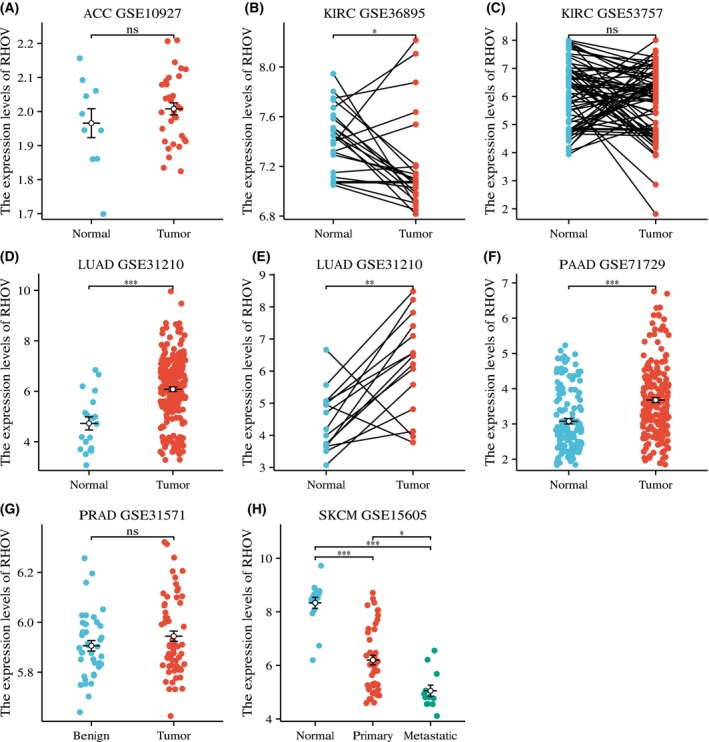
Validation of *RHOV* expression patterns in adrenocortical carcinoma (ACC), kidney renal clear cell carcinoma (KIRC), lung adenocarcinoma (LUAD), pancreatic adenocarcinoma (PAAD), prostate adenocarcinoma (PRAD), and skin cutaneous melanoma (SKCM). (A) ACC GSE10927. Normal (*n* = 10), Tumor (*n* = 33). (B) KIRC GSE36895 (*n* = 23). (C) KIRC GSE53757 (*n* = 72). (D) LUAD GSE31210. Normal (*n* = 20), Tumor (*n* = 226). (E) LUAD GSE31210 matched samples (*n* = 15). (F) PAAD GSE71729. Normal (*n* = 134), Tumor (*n* = 204). (G) PRAD GSE31571. Benign (*n* = 39), Tumor (*n* = 59). (H) SKCM GSE15605. Normal (*n* = 16), Primary (*n* = 46), Metastatic (*n* = 12). (Wilcoxon rank‐sum test. ns, *P* > 0.05; *P < 0.05; **P < 0.01; ***P < 0.001).

Based on the prognostic value of *RHOV* in the analyses described above, we further attempted to validate the expression pattern of *RHOV* in these tumors by exploring GEO datasets. *RHOV* expression was relatively low in ACC samples, with no differential expression between tumor and normal tissues (Fig. 8A). In KIRC, the GSE36895 dataset showed downregulated expression of *RHOV* in tumors compared with normal tissues (Fig. 8B), which was consistent with the results from TCGA_GTEx, whereas the GSE53757 dataset showed no differential expression between matched tumor and normal tissues (Fig. 8C). *RHOV* expression was upregulated in both LUAD (Fig. 8D,E) and PAAD (Fig. 8F), confirming the results from TCGA_GTEx. However, no differential expression was observed for PRAD in the GSE32571 dataset (Fig. 8G). In SKCM, *RHOV* expression was downregulated in primary and metastatic tissues compared with normal tissues, and it was also downregulated in metastatic tissues compared with primary tissues (Fig. 8H). No GEO series of UVM‐containing normal tissues could be identified for this analysis.

### Correlation between *RHOV* expression and clinicopathological parameters

**Fig. 9:**
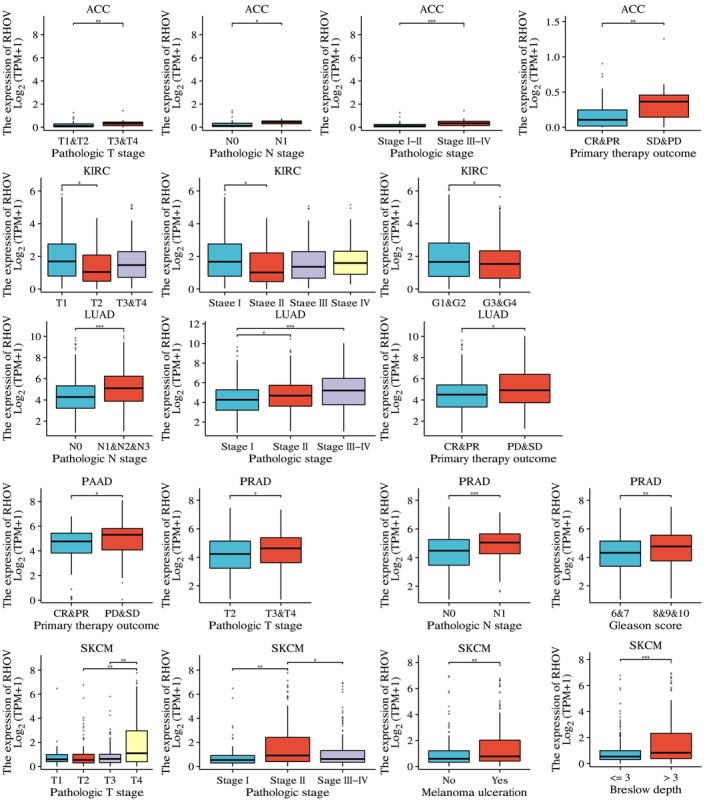
Correlations between *RHOV* expression and clinicopathological parameters in seven types of cancers in which *RHOV* expression was associated with prognosis. Only significant features are shown. ACC: T1&T2 (*n* = 51), T3&T4 (*n* = 26); N0 (*n* = 68), N1 (*n* = 9); stage I&II (*n* = 46), stage III&IV (*n* = 31); CR&PR (*n* = 47), SD&PD (*n* = 20). KIRC: T1 (*n* = 279), T2 (*n* = 71), T3&T4 (*n* = 191); stage I (*n* = 273), stage II (*n* = 59), stage III (*n* = 123), stage IV (*n* = 83); G1&G2 (*n* = 250), G3&G4 (*n* = 283). LUAD: N0 (*n* = 350), N1&N2&N3 (*n* = 173); stage I (*n* = 296), stage II (*n* = 125), stage III&IV (*n* = 110); CR&PR (*n* = 340), SD&PD (*n* = 109). PAAD: CR&PR (*n* = 81), SD&PD (*n* = 59). PRAD: T2 (*n* = 189), T3&T4 (*n* = 305); N0 (*n* = 348), N1 (*n* = 80); Gleason score 6&7 (*n* = 294), 8&9&10 (*n* = 207). SKCM: T1 (*n* = 42), T2 (*n* = 79), T3 (*n* = 91), T4 (*n* = 153); stage I (*n* = 78), stage II (*n* = 140), stage III&IV (*n* = 195); ulceration No (*n* = 148), Yes (*n* = 167); Breslow depth ≤ 3 (*n* = 186), > 3 (*n* = 175). (Wilcoxon rank‐sum test. *P < 0.05; **P < 0.01; ***P < 0.001).

We further investigated the association between *RHOV* expression and various clinicopathological parameters in the six tumors for which *RHOV* demonstrated prognostic significance and relevant data were available. High *RHOV* expression correlated with high pathological T stage in ACC, PRAD, and SKCM; high pathological N stage in ACC, LUAD, and PRAD; advanced pathological stage in ACC, LUAD, and SKCM; and worse treatment outcomes in ACC, LUAD, and PAAD. High *RHOV* expression was also associated with a high Gleason score in PRAD, as well as with the presence of ulceration and Breslow depth in SKCM (Fig. 9).

### Correlation of *RHOV* expression with TME

**Fig. 10:**
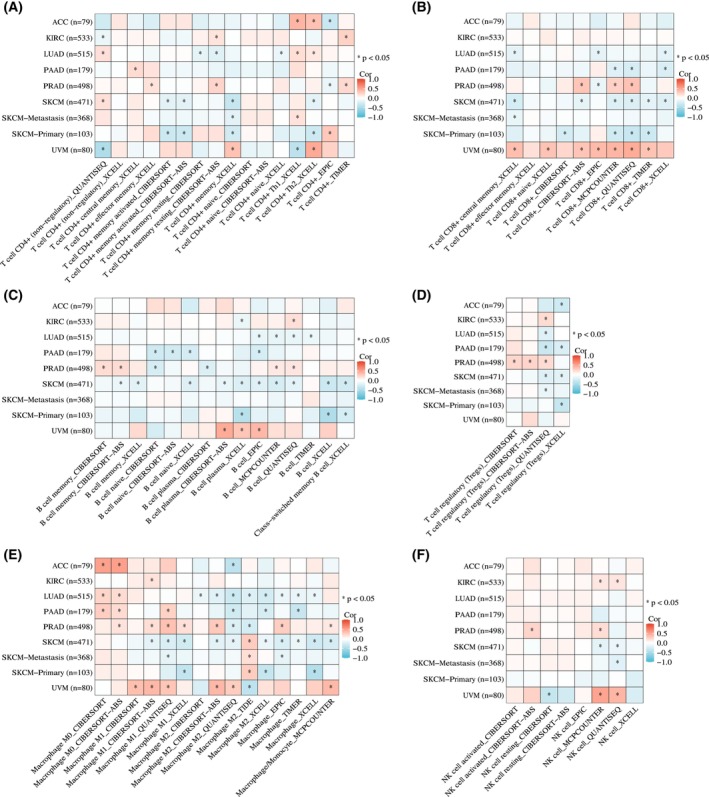
Heatmaps showing the correlation of *RHOV* expression with immune cell infiltration in the TIMER 2.0 database. (A) CD4+ T cells. (B) CD8+ T cells. (C) B cells. (D) Regulatory T cells (Tregs). (E) Macrophages. (F) Natural killer (NK) cells.

**Fig. 11:**
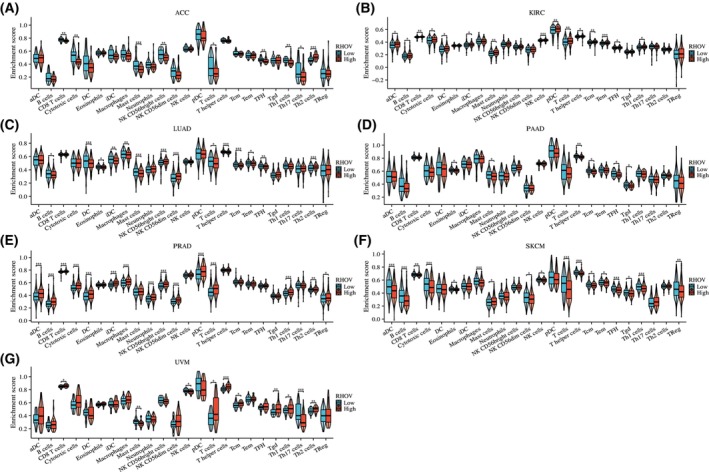
Correlation between *RHOV* expression and 24 types of intratumor immune cells in seven types of cancers in which *RHOV* expression was associated with prognosis. (A) ACC (*n* = 79). (B) KIRC (*n* = 541). (C) LUAD (*n* = 539). (D) PAAD (*n* = 179). (E) PRAD (*n* = 501). (F) SKCM (*n* = 472). (G) UVM (*n* = 80). (Wilcoxon rank‐sum test. *P < 0.05; **P < 0.01; ***P < 0.001).

**Fig. 12:**
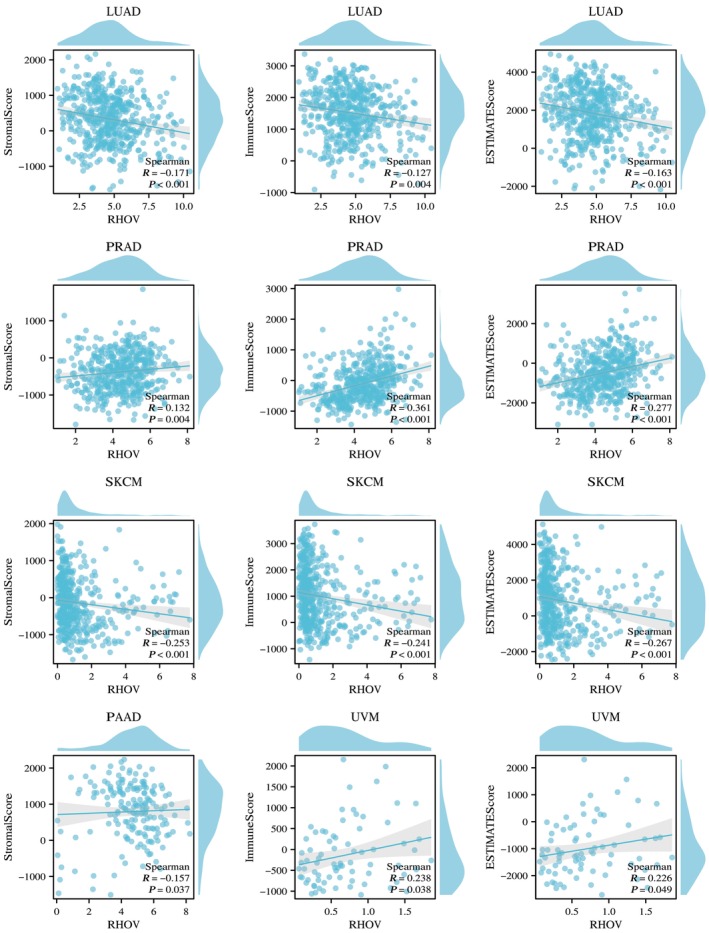
Correlation between RHOV expression and ImmuneScore, StromalScore, as well as ESTIMATEScore in seven types of cancers.

Heatmaps in Fig. 10 demonstrate correlations between *RHOV* expression and CD4+ T cells, CD8+ T cells, B cells, regulatory T cells, macrophages, and NK cells. *RHOV* expression was negatively correlated with CD8+ T and B cells in LUAD and SKCM, whereas it was significantly correlated with various immune cells in PRAD and UVM. In ACC and SKCM, *RHOV* expression was negatively correlated with T cells, CD8+ T cells, and cytotoxic cells, whereas positive correlations for these cells were observed in KIRC and PRAD tumors. *RHOV* expression also correlated negatively with B cells, DCs, and induced DCs in LUAD, but positively correlated with these cells in KIRC and PRAD (Fig. 11). Furthermore, *RHOV* expression was negatively correlated with ImmuneScore and ESTIMATEScore in LUAD and SKCM but positively correlated with these scores in PRAD and UVM (Fig. 12).

### Correlation of *RHOV* expression with response to ICIs

**Fig. 13:**
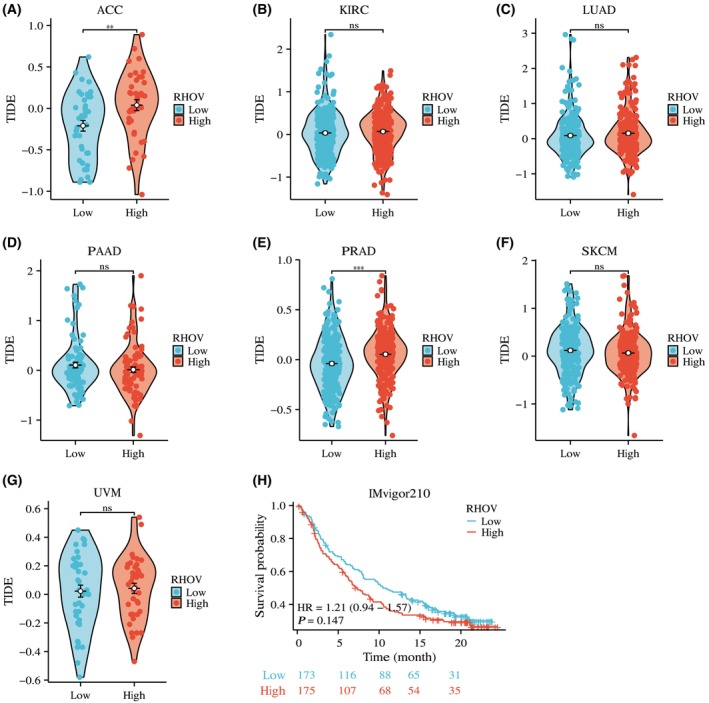
Correlation of *RHOV* expression level with response to immune checkpoint inhibitors (ICIs). (A–F) Comparison of TIDE scores between patients with low and high *RHOV* expression levels in seven types of cancers. ACC (*n* = 79). KIRC (*n* = 532). LUAD (*n* = 522). PAAD (*n* = 178). PRAD (*n* = 483). SKCM (*n* = 469). UVM (*n* = 77) (H) Survival analysis between patients with low and high *RHOV* expression levels in the IMvigor210 cohort (*n* = 348). (A–G: Wilcoxon rank‐sum test. H: log‐rank test. ns, *P* > 0.05; **P < 0.01; ***P < 0.001).

Based on the correlation between *RHOV* expression and immune infiltration, we further explored whether *RHOV* expression can predict the potential benefits of ICIs. As shown in Fig. 13, for ACC (Fig. 13A) and PRAD (Fig. 13E), the TIDE score was significantly higher in the high *RHOV* expression group than in the low *RHOV* expression group, suggesting that high *RHOV* expression levels may predict worse responses to ICIs in these tumors. However, in KIRC (Fig. 13B), LUAD (Fig. 13C), PAAD (Fig. 13D), SKCM (Fig. 13F), and UVM (Fig. 13G), the TIDE score was similar between the high and low *RHOV* expression groups, indicating that the *RHOV* expression level would not be a useful predictor of the benefits of ICIs in these tumors. Analysis of the IMvigor210 cohort showed no difference in survival between the high and low *RHOV* expression groups (Fig. 13H).

### Functional enrichment analysis of *RHOV* ‐related genes

**Fig. 14:**
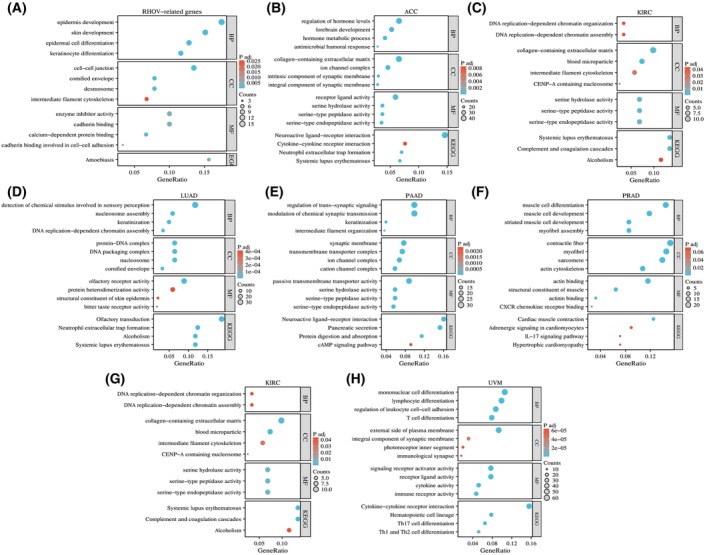
Gene Ontology (GO) and Kyoto Encyclopedia of Gene and Genomes (KEGG) functional enrichment analyses of *RHOV*‐related genes. (A) Top‐100 genes with similar expression patterns to *RHOV* in pan‐cancer. (B–H) Differentially expressed genes between high and low *RHOV* expression groups in seven types of cancers in which *RHOV* expression was associated with prognosis.

GO analysis suggested that *RHOV*‐related genes may be involved in various biological processes (Table 9), including ‘epidermis development,’ ‘regulation of hormone levels,’ ‘forebrain development,’ ‘DNA‐replication‐dependent chromatin organization,’ ‘detection of chemical stimulus involved in sensory perception,’ ‘intermediate filament organization,’ ‘muscle cell differentiation,’ and ‘mononuclear cell differentiation.’ KEGG analysis indicated that *RHOV*‐related genes may participate in pathways such as ‘amoebiasis,’ ‘neuroactive ligand‐receptor interaction,’ ‘cytokine‐cytokine receptor interaction,’ ‘complement and coagulation cascades,’ ‘systemic lupus erythematosus,’ ‘cAMP signaling pathway,’ ‘IL‐17 signaling pathway,’ ‘Th 17 cell differentiation,’ and ‘Th1 and Th2 cell differentiation’ (Fig. 14).

**Table 9:** Top‐100 genes of most similar expression pattern with RHOV in pan‐cancer.

Gene symbol	PCC	Gene symbol	PCC	Gene symbol	PCC
AIM1L	0.6	SDC1	0.5	TRIM7	0.45
TRIM29	0.6	FABP5	0.5	TMEM79	0.45
PPP1R13L	0.59	RHBDL2	0.5	FAM83C	0.45
PVRL4	0.58	HCAR2	0.49	PROM2	0.45
GPR87	0.58	GRHL1	0.49	SERPINB5	0.45
GJB5	0.57	PKP3	0.49	PLEKHN1	0.45
HES2	0.57	PKP1	0.49	PRRG4	0.45
S100A16	0.57	CERS3	0.49	CSTA	0.45
TACSTD2	0.56	ANXA8L1	0.48	ZNF185	0.45
ZNF750	0.56	DSC3	0.48	STARD5	0.45
S100A11	0.55	DSG3	0.48	TYMP	0.45
LAD1	0.55	FABP5P7	0.48	PRSS22	0.44
IRF6	0.55	FAM83A	0.48	KRT17	0.44
PERP	0.55	IPPK	0.48	RAPGEFL1	0.44
MIR205HG	0.54	EVPL	0.48	DSP	0.44
S100A14	0.54	S100A8	0.48	ALG1L	0.44
LY6D	0.53	GSDMC	0.48	SERPINB4	0.44
FAT2	0.53	ANXA8	0.48	GRHL3	0.43
REEP4	0.53	S100A2	0.48	HCAR3	0.43
GJB3	0.52	SERPINB3	0.48	DUSP7	0.43
LYPD3	0.52	TMEM40	0.47	GPR157	0.43
SFN	0.52	CSTB	0.47	C1orf74	0.43
IL20RB	0.52	TMPRSS11D	0.47	ITPKC	0.43
GNA15	0.52	SEMA4B	0.47	MICALL1	0.43
ADGRF4	0.52	CTA‐384D8.35	0.47	FXYD3	0.42
DENND2C	0.52	FAM110A	0.47	AC005262.3	0.42
PITX1	0.51	SERPINB13	0.47	SCO2	0.42
HSPB1	0.51	RARG	0.46	HNRNPA1P33	0.42
S100A9	0.51	PGLYRP3	0.46	CDKN2B	0.42
EPHX3	0.51	IER5	0.46	ARPC5L	0.42
PVRL1	0.51	DTX2	0.46	GLTP	0.42
KRT5	0.51	CAPNS2	0.46	OVOL1	0.42
S1PR5	0.5	ATP1B3	0.46	RAB25	0.42
RP3‐523K23.2	0.5				

**Fig. 15:**
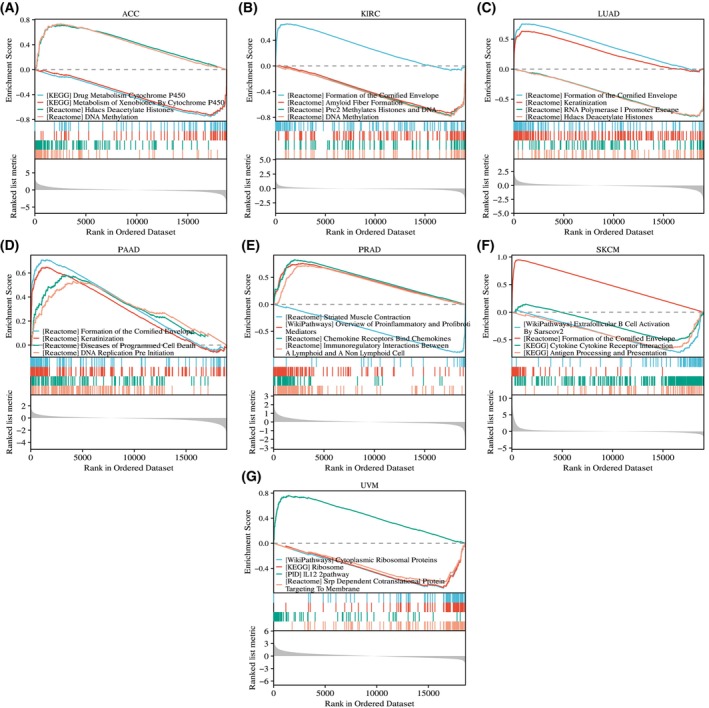
Gene set enrichment analysis of differentially expressed genes between high and low *RHOV* expression groups in seven types of cancers for which *RHOV* expression was associated with prognosis. (A) ACC. (B) KIRC. (C) LUAD. (D) PAAD. (E) PRAD. (F) SKCM. (G) UVM.

GSEA of the seven tumors for which *RHOV* had prognostic significance provided further evidence of the potential biological functions of *RHOV*, including roles in ‘drug metabolism cytochrome p450,’ ‘DNA methylation,’ ‘amyloid fiber formation,’ ‘formation of the cornified envelope,’ ‘diseases of programmed cell death,’ ‘immunoregulatory interactions between a lymphoid and a non‐lymphoid cell,’ and ‘cytokine‐cytokine receptor interaction’ (Fig. 15).

## Discussion

In this comprehensive study, the expression and prognostic role of RHOV in pan‐cancer were thoroughly investigated. The results revealed that *RHOV* expression was upregulated in 18 tumor types and downregulated in seven tumor types. The association between *RHOV* expression and prognosis varied across different tumor types. Specifically, in KIRC and PRAD, in which *RHOV* expression was downregulated, higher *RHOV* expression was associated with a more favorable prognosis. In contrast, *RHOV* expression was associated with an unfavorable prognosis in ACC, LUAD, PAAD, SKCM, and UVM. *RHOV* expression was upregulated in LUAD and PAAD and was downregulated in SKCM. ACC exhibited low overall expression of *RHOV*, and differential expression was not available for UVM. These findings suggest that *RHOV* may have diverse functions, acting either as an oncogene or as a tumor suppressor, depending on the specific tumor and tumor microenvironment.

Previous studies demonstrated that *RHOV* functions as an oncogene in LUAD, promoting cancer cell growth, metastasis, and resistance to epidermal growth factor receptor (EGFR)‐tyrosine kinase inhibitor therapies [21, 22, 23]. RHOV has been demonstrated to be a pro‐metastatic factor in TNBC [24]. However, evidence supporting the tumor‐suppressive role of RHOV remains unavailable. Notably, RHOV showed potential to induce apoptosis in PC12 cells (a rat pheochromocytoma cell line) and RAW264.7 macrophages [19, 20], suggesting its potential to induce apoptosis in cancer cells. However, further investigation is warranted in this regard.

Results from the immune infiltration analysis suggested that *RHOV* expression correlated with various immune cell types across the analyzed tumors. In addition, *RHOV* expression correlated with ImmuneScore, StromalScore, and ESTIMATEScore in LUAD, SKCM, PRAD, and UVM. However, this correlation was inconsistent among different cancers, suggesting that the influence of RHOV on the immune system may be specific to individual cancer types. Although high *RHOV* expression levels in ACC and PRAD predicted lower responses to ICIs, considering the complexity of the TME and the limitation of a single gene as a biomarker to predict potential benefits from ICIs, we do not consider RHOV as a useful clinical marker of the response to ICIs at this point.

Functional enrichment analyses of *RHOV*‐related genes revealed several important biological processes and pathways that could shed light on the potential mechanisms underlying the correlation between RHOV, cancer prognosis, and immune infiltration. One notable finding was the enrichment of terms related to cell migration, such as ‘cell–cell junction,’ ‘intermediate filament cytoskeleton,’ ‘cadherin binding involved in cell–cell adhesion,’ and ‘collagen‐containing extracellular matrix.’ These findings are consistent with previous studies demonstrating the role of RHOV in regulating cell adhesion and promoting cell movement in various contexts, including zebrafish epiboly and cancer metastasis [18]. Furthermore, RHOV activates the Jun N‐terminal kinase/c‐Jun signaling pathway, which has been implicated in LUAD cell growth and metastasis [23]. Additionally, RHOV activates EGFR signaling through GRB2, thereby regulating cancer cell migration and promoting metastasis in TNBC [24]. The *RHOV*‐related genes were enriched in various immune‐related pathways, including ‘cytokine–cytokine receptor interaction,’ ‘antigen processing and presentation,’ ‘IL‐17 signaling pathway,’ and ‘IL‐12 pathway.’ These findings suggest a potential correlation between RHOV and the immune response. Although one study suggested the involvement of RHOV in the differentiation of myeloid cells [26], the detailed mechanism remains unknown. Furthermore, there is currently no experimental evidence to directly support the effect of RHOV on TIICs, highlighting the need for further investigation in this area.

Our study used *in silica* data mining to explore the prognostic and immunological roles of RHOV in various cancer types. The results of our study indicated a dual effect of RHOV on prognosis and immune infiltration. Although we tried our best to strengthen our results, some limitations of this type of analysis are inevitable. First, although we observed different prognostic roles for *RHOV* in different cancers, we were unable to provide experimental evidence to confirm whether *RHOV* acts as an oncogene or a tumor suppressor in specific cancer types. Second, although we found associations between *RHOV* expression and immune infiltration and identified immune‐related pathways through enrichment analysis, there is currently no clear evidence to support the notion that *RHOV* directly influences the immune cell composition of the TME. Third, the exact mechanism by which RHOV affects the survival of patients with cancer through the regulation of TIICs remains unknown.

In conclusion, our study revealed the dysregulation of RHOV expression in various tumors and identified its prognostic significance in several cancer types. Additionally, *RHOV* expression correlated with the distribution of different TIICs in these cancers. However, further experimental investigations are required to validate the functional roles of RHOV in specific cancer types and its impact on TIICs and patient survival.

## Conflict of interest

The authors declare no conflict of interest.

### Peer review

The peer review history for this article is available at https://www.webofscience.com/api/gateway/wos/peer‐review/10.1002/2211‐5463.13698.

## Author contributions

QQ and BP designed and performed the study, and collectively wrote and reviewed the manuscript.

## Data Availability

Publicly available datasets were analyzed in this study; further inquiries can be directed to the corresponding author.
